# Dental safety of short-term doxycycline use in children under 8 years: a systematic review and meta-analysis

**DOI:** 10.3389/fphar.2025.1646638

**Published:** 2025-09-24

**Authors:** Anusiga Sigamani Rajan, Muthu Gopal, Madhumidha Periyathambi, Vijesh Sreedhar Kuttiatt

**Affiliations:** ^1^ Model Rural Health Research Unit, Puducherry, India; ^2^ Unit of Clinical and Molecular Medicine, ICMR-Vector Control Research Centre, Puducherry, India

**Keywords:** adverse event, antibiotics, children under 8 years, dental safety, doxycycline

## Abstract

**Background:**

Doxycycline is widely used to treat various bacterial infections and has significant global implications, especially as the preferred medication for scrub typhus and leptospirosis. However, its use is limited in children below 8 years of age due to potential adverse effects on teeth and bones. This study aims to assess the risk of dental adverse events associated with doxycycline, a tetracycline-class antibiotic, in children under 8 years of age.

**Methods:**

In this review, we searched PubMed, the Cochrane Library, and Google Scholar for studies published until November 2023. We included studies administering doxycycline to children under 8 years, focusing on dose, duration, and dental adverse events to assess the safety of doxycycline.

**Result:**

A total of 325 articles were initially retrieved, of which 5 studies were available for analysis. This review included information on 162 children who were treated with doxycycline between the ages of 0 and 8 years. The median age at doxycycline administration was 4.25 years and interquartile range (IQR) of 2.065–5.563 years. The oral dosage, based on weight, had a median of 2.3 mg/kg/day, with an IQR of 1.525–5.438 mg/kg/day. The median duration of drug administration was 8.5 days and the IQR of 6–12.5 days. We found a pooled proportion of adverse events of 0.21 (95% CI: 0.13–0.28).

**Conclusion:**

This review suggests that the occurrence of teeth-related adverse events with short-term doxycycline use is minimal, with a low incidence reported. While these findings offer a preliminary basis for the use of doxycycline in children under 8 years of age, the limited number of studies underscore the need for further research to evaluate its therapeutic use and implication for paediatric guidelines.

**Systematic Review Registration:**

https://www.crd.york.ac.uk/PROSPERO/myprospero, Identifier CRD42023494713.

## Introduction

Doxycycline is a commonly used antibiotic that belongs to the class of tetracyclines. It was developed in 1967 and has been widely used since then. Doxycycline is effective against both the Gram-negative and Gram-positive bacteria and is used to treat infections of the respiratory tract, skin and for sexually transmitted diseases such as syphilis, *chlamydia* and pelvic inflammatory disease. Doxycycline is effective against undifferentiated febrile illnesses like brucellosis, leptospirosis, scrub typhus and other rickettsial illnesses (Ruhe and Menon, 2007). It can be used for the treatment of Lyme disease, tularemia, plague and cholera and for chemoprophylaxis against malaria. Remarkably, it is effective against methicillin-resistant *Staphylococcus aureus* (MRSA) as well.

The dose and duration of doxycycline treatment depend on the specific conditions being treated, as well as the patient’s age, weight, and overall health. Reported side effects of doxycycline include gastrointestinal complaints, photosensitivity, and, in rare cases, allergic reactions. Doxycycline is generally not recommended for children under 8 years, when used for prolonged treatment or repeated courses, due to potential adverse effects on bones and teeth (Wormser et al., 2019b). However, the American Academy of Pediatrics recently updated its guidelines, stating that while tetracyclines are contraindicated for common infections in children younger than 8 years, doxycycline is recommended as the drug of choice for treating presumed or confirmed Rocky Mountain spotted fever (RMSF) in children of any age (Red Book: AAP, 2021). Despite this, the use of doxycycline in young children presents a clinical dilemma due to the limited number of studies on adverse events and the lack of strong evidence-based supporting data for recent clinical recommendations. This review aims to critically assess the risk of dental adverse events following doxycycline and its average course in children under 8 years of age.

## Methods

This review was initiated following registration with the International Prospective Register of Systematic Reviews (PROSPERO). The review was performed in accordance with the reporting requirements outlined in the “Preferred Reporting Items for Systematic Reviews and Meta-Analyses (PRISMA) guidelines.”

### Eligibility criteria

The inclusion criteria, based on the PICOT(S) format, targeted studies involving children under 8 years of any ethnicity or sex, treated with any dose of doxycycline alone or with other drugs. Comparators included placebo or other antibiotics. Outcomes had to report on safety or adverse events, particularly dental effects. Any treatment duration was accepted, but at least 1 year of follow-up was required. Eligible study designs included RCTs, cohort, case-control, or clinical trials in hospital or community settings.

Other broad inclusion and exclusion criteria are provided in the Supplementary Material.

### Search strategy

A systematic literature review was conducted using a combination of Medical Subject Headings (MeSH) terms in PubMed, Cochrane Library, and Google Scholar for retrieving studies from inception till November 2023. www.clinicaltrials.gov was also searched. The above-mentioned databases were searched using ‘doxycycline’ alone or in combination with ‘Paediatric’ OR ‘children’ OR ‘safety’ OR ‘adverse event’. The search strategies used for PubMed and Cochrane Library are provided in the Supplementary Material. The PROSPERO registration number is “CRD42023494713.”

### Selection of studies

The search results were uploaded into Covidence, for selection of studies. A two-stage screening process was carried out. The titles and abstract screening were conducted by two reviewers (ASR and MP) independently. The full texts of the articles were obtained for the relevant records and independently reviewed by two reviewers (ASR and MP). Any discrepancies were resolved through discussion, involving a senior reviewer (GM) if necessary.

### Data extraction and management

The two reviewers (ASR and MP) independently extracted the pre-defined study characteristics, baseline data, and the safety outcomes from the included studies using a data extraction form (Supplementary Material).

### Data analysis

Data were analyzed using Jamovi (version 2.4.14 for Windows). Proportions were pooled with 95% confidence intervals (CI), using a meta-analysis model that applied the Freeman–Tukey double arcsine transformation to stabilize variance. The random-effects model was used in cases of significant heterogeneity; otherwise, the fixed-effect model was employed.

### Quality assessment

Two independent reviewers (ASR and MP) evaluated the potential bias for all the included studies utilizing the criteria from the Revised Cochrane risk-of-bias tool (RoB2) for randomized trials (Cochrane Methods Bias, 2025) and JBI Critical Appraisal tools and checklist were used to assess the risk of bias for case-control and cohort studies (JBI, 2025).

## Results

**FIGURE 1:**
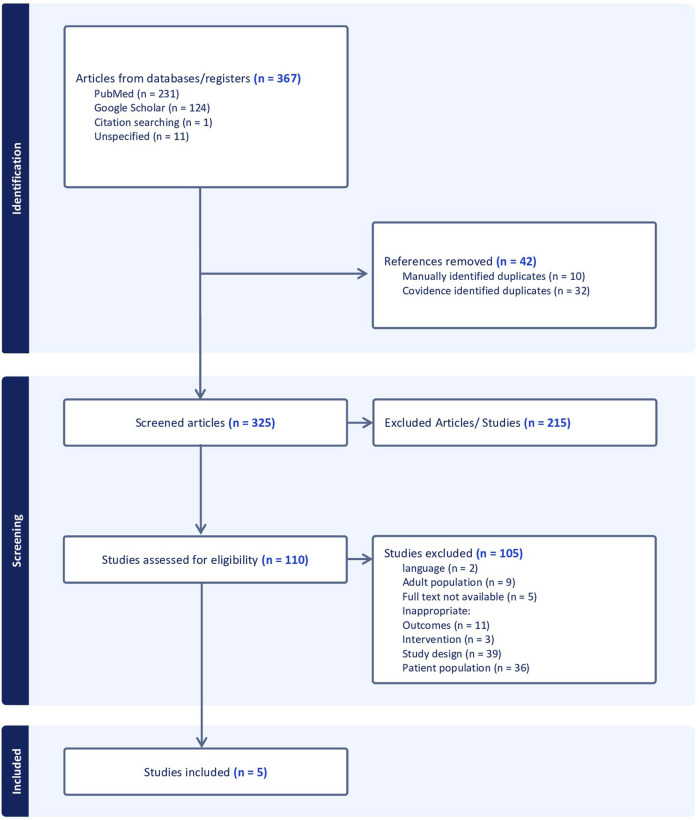
PRISMA flow diagram.

Of the 325 deduplicated data, only five studies met the inclusion criteria. (All the detailed information is provided in the PRISMA diagram (Figure 1)). The characteristics of the five studies were summarized in Table 1. Forti and Benincori, (1969), Lochary et al. (1998), Volovitz et al. (2007), Todd et al. (2015), Pöyhönen et al. (2017).

**TABLE 1:** Summary of the key elements of included studies.

Author, Year and Country	Disease conditions under study	Study design	Sample size^a^	Age at exposure	Age at dental examination	Dose, route and duration	Dental assessment method	Adverse events of doxycycline	Risk of Bias
Todd SR et al. (2015), United States	Rocky Mountain spotted fever (RMSF)	Hospital-based retrospective cohort study	58:213	0.2–7.9 years	8.1–15.6 years	Dose: 2.3 mg/kg with SD of 0.40 (range 0.3–2.9) Route: Oral (98%) Duration: 7.3 days with SD of 2.8 (range 1–10); BID daily (97%)	Dental examination with VITA Easy shade Compact instrument (Vita Zahnfabrik H. Rauter GmbH and Co., BadSâ‚¬ackingen, Germany), a handheld spectrophotometer used to evaluate the shade of teeth	Tetracycline-like staining was not detected Enamel hypoplasia - 2 (4%)	Low
Lochary ME et al. (1998), United Kingdom	Rocky Mountain spotted fever	Hospital-based retrospective case-control study	10:20	4.3–8.25 years	11–19 years	Dose: 30 mg–200 mg Route: First IV and later oral in one child, only oral in 8 children Duration: BID daily for 7–10 days	The teeth were photographed using a NikonÂ^®^ 35-mm intraoral camera with both ring and point flash, and Kodak 100Â^®^ speed print film Staining was rated by 5 dentists on a scale of 0 (normal) to 3 (severe discoloration)	No tooth staining was detected	Moderate
Pöyhönen et al. (2017), Finland	Suspected acute central nervous system infection with *Borrelia burgdorferi* and *Mycoplasma pneumoniae*	Hospital-based retrospective cohort study	38	0.6–7.9 years	8.3–22.6 years	Dose: 10 mg/kg/day loading dose for the first two to 3 days, with an average dose of 5 mg/kg/day with a range of 2.5–10 mg/kg/day Route: IV first, then oral in 18; in 12 children, solely oral, and in 9 children, just IV (one received two courses of doxycycline) Duration: 12.5 days (range two to 28 days, SD 6)	Teeth were photographed using a mouth mirror from five perspectives with a Canon 7D camera (Canon, Tokyo, Japan) and examined by two independent paediatric dentists	No staining resembling that caused by tetracycline or enamel hypoplasia was observed	Moderate
Volovitz B et al. (2007), Israel	uncontrolled asthma believed to be caused by an infection with *M pneumoniae*	Single-blinded, randomised, controlled study	31:30	2–7.7 years	8–16 years	Dose: On day one, 4 mg/kg BID followed by an OD of 2 mg/kg/day for 9 days Route: Oral syrup Duration: 10 days	The examination was conducted using visual inspection with a dental unit light, dental explorer, and mirror Tooth color was assessed using the Lumin Vacuum Shade Guide, which includes 16 shades ranging from lighter (white) to darker (gray)	No tooth staining was detected	Moderate
Forti and Benincori, (1969), Italy	NA	NA	25	4–55 days	One year after treatment	Dose: 2 mg/kg/day for the 1st day followed by 1 mg/kg/day Route: NA Duration: 6–17 days	The teeth were examined through direct observation and by fluorescence using Wood’s light	One child developed discoloration in the upper incisor, duration of the discoloration not reported	Critical

^a^ In case both groups as intervention and control - given as I:C. NA, not available.

The included studies were published between 1969 and 2017. All five studies were conducted in hospital settings. Of these, four were retrospective in nature (two were cohort studies, one was a case-control study, and the fourth was a retrospective study with unspecified design) while the fifth was a single-blinded, randomized controlled study. In the selected five studies, a total of 162 children less than 8 years of age received treatment with doxycycline for various indications. The disease conditions were Rocky Mountain spotted fever (RMSF) (n = 68), suspected acute central nervous system infection with *Borrelia burgdorferi* or *Mycoplasma pneumoniae* (n = 38), uncontrolled asthma believed to be caused by an infection with *M pneumoniae* (n = 31) and for premature infants to treat infections (n = 25). Doxycycline was administered orally (n = 109), intravenous (n = 9), first intravenous than orally (n = 19).

Data extracted from the five studies identified in this systematic review included information on 162 children who were treated with doxycycline between the ages of 0 and 8 years for various medical indications. The median age at doxycycline administration was 4.25 and IQR of 2.065–5.563. The oral dosage, based on weight, had a median of 2.3 mg/kg/day, with an IQR of 1.525–5.438 mg/kg/day. The median duration of drug administration was 8.5 days and IQR 6–12.5 days.

Heterogenicity test: A Q-statistic of 0.954 with a p-value of 0.917 and an I^2^ of 0% indicates low heterogeneity across studies, so a fixed-effect model is used.

### Adverse events

**FIGURE 2:**
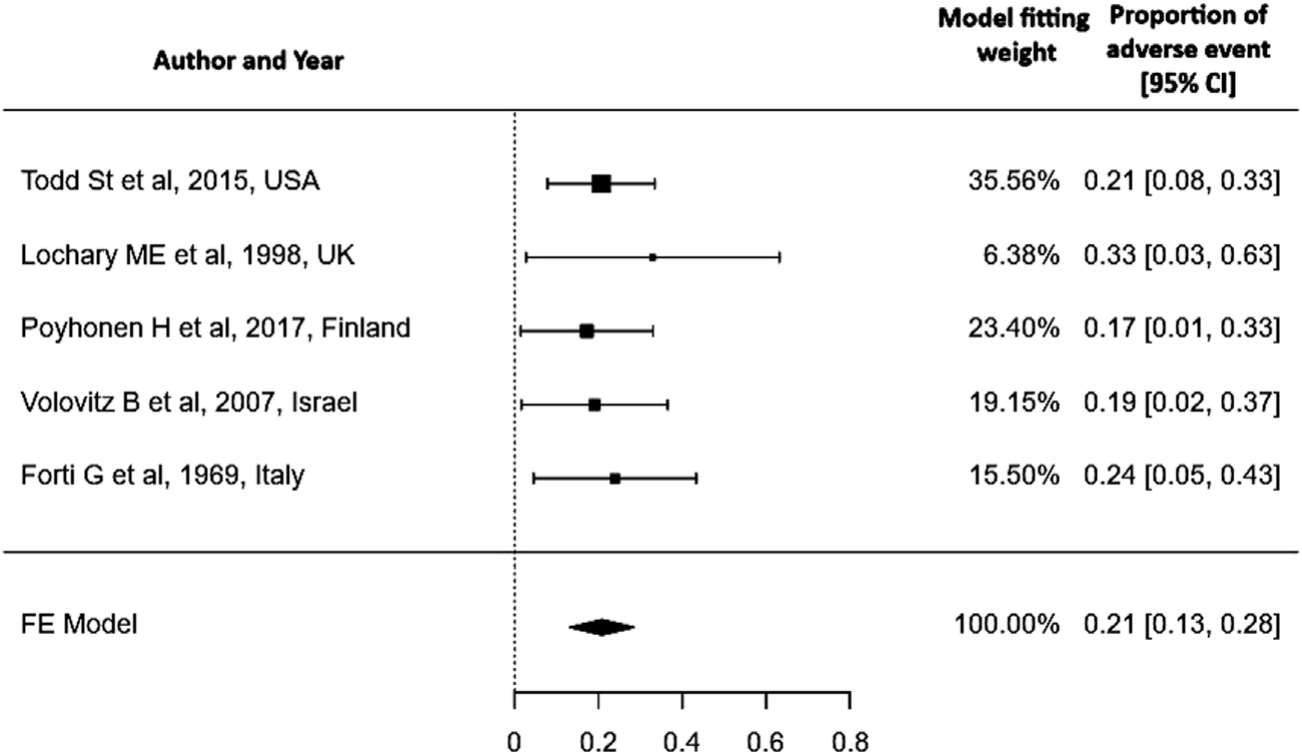
Pooled proportion of adverse events.

The included studies specifically examine the adverse effects of doxycycline on dentition, providing information exclusively on its impact in this area (tooth discoloration, enamel hypoplasia) without addressing other adverse events (e.g., gastrointestinal symptoms, photosensitivity, allergic reactions) hence this review does not provide evidence on the overall safety of doxycycline in children under 8 years of age. Out of 162 children, only one premature infant (1/162) had tooth discoloration of the upper incisors (0.62%) and two other children had enamel hypoplasia (1.23%). A meta-analysis of proportions was conducted using the Freeman–Tukey double arcsine transformation to stabilize variance. A fixed-effect model was applied to pool data from five studies. The pooled proportion of the adverse event was 0.21 (95% CI: 0.13–0.28). A fixed-effect model was chosen due to negligible heterogeneity among studies (I^2^ = 0%, p = 0.917) Figure 2. Although the overall risk of bias varied across studies, evidence from three low-risk-of-bias studies indicated that doxycycline did not cause dental staining (Lochary et al., 1998; Pöyhönen et al., 2017; Volovitz et al., 2007).

### Publication bias assessment

**FIGURE 3:**
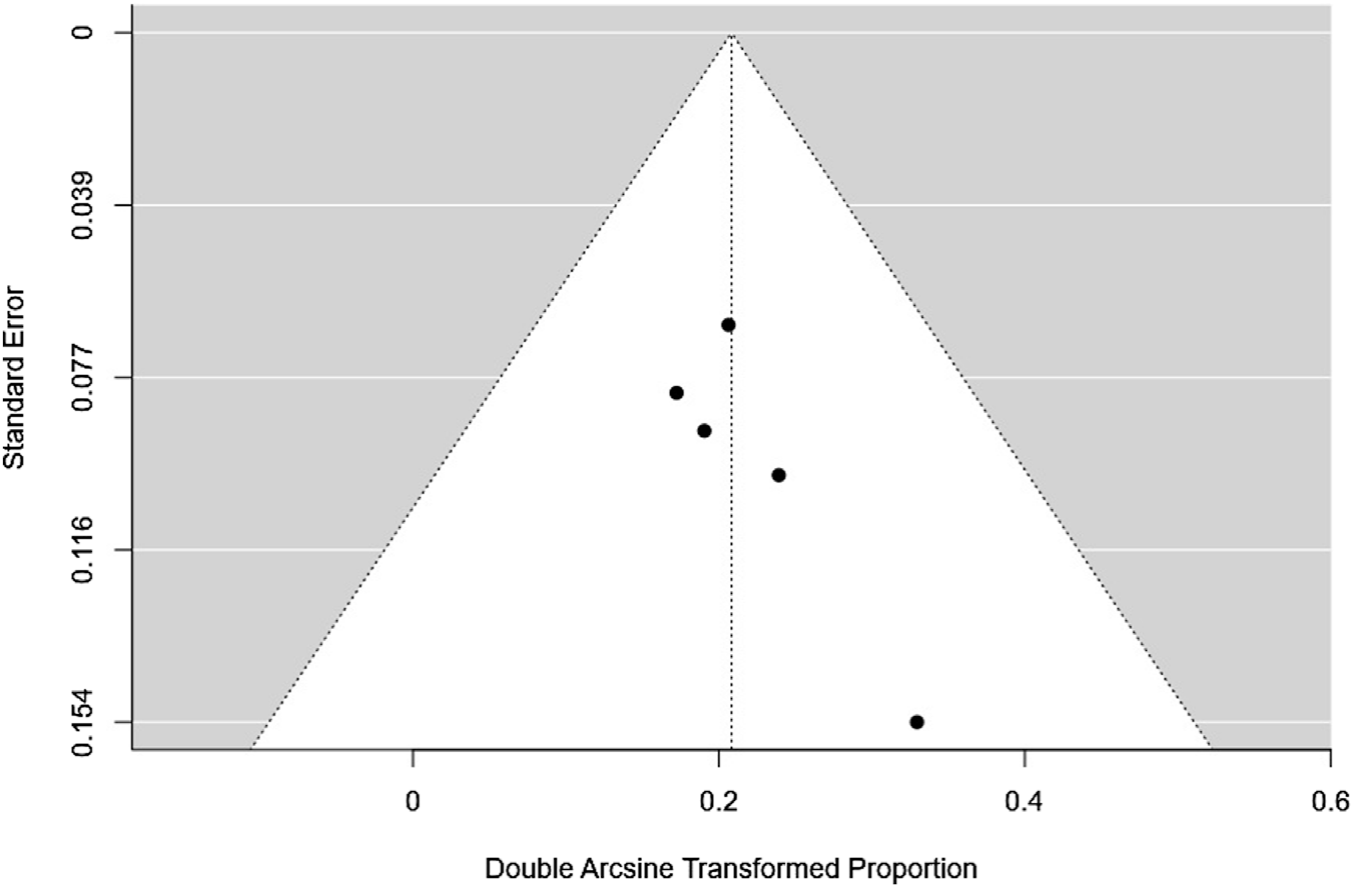
Funnel plot.

The fail-safe N was 49 (p < 0.001), indicating that 49 additional null-result studies would be needed to nullify the observed effect. Both the rank correlation test (Kendall’s Tau = 0.600, p = 0.233) and the regression test (Z = 0.749, p = 0.454) were non-significant, suggesting no notable funnel plot asymmetry. Overall, these results indicate minimal evidence of publication bias and support the reliability of the observed small effect size Figure 3.

## Discussion

Doxycycline is widely used to treat various bacterial infections and has significant global implications, especially as the preferred medication for scrub typhus and leptospirosis. However, its use is limited in children below 8 years of age due to potential adverse effects on teeth and bones. Since 1958, the use of tetracycline has been linked to tooth discoloration in children (Shwachman et al., 1958), with subsequent studies confirming this association (Shwachman et al., 1958; Wallman and Hilton, 1962; Porter et al., 1965). These studies primarily recommend avoiding tetracyclines, such as doxycycline, in young children. Yet, recent high-quality evidence-based data remain scarce, making it challenging to draw definitive conclusions regarding the safety of doxycycline use in children. Recognizing this gap, the present review was conducted to systematically evaluate the available literature. Among 162 children who received doxycycline before the age of 8, only one premature infant less than 2 months old, exhibited discoloration in a deciduous tooth. Children with permanent teeth show no discoloration (n = 137) and their median age at dental examination was 13.5 and IQR of 11.89–15.34 years. Similarly, a study revealed that permanent teeth are less affected by tetracycline staining compared to primary teeth (Grossman et al., 1971). This better outcome of the doxycycline may be attributed to noticeably different pharmacokinetic properties, which allow for less frequent administration and lower total daily doses of doxycycline (Wormser et al., 2019a). In addition, when compared to other tetracyclines, doxycycline exhibits a lower affinity for binding to calcium, with a binding rate of 19% for doxycycline *versus* 39.5% for tetracycline (Forti and Benincori, 1969).

Administering short courses of doxycycline, lasting 10 days or less, to children under 8 years for the treatment of Rocky Mountain Spotted Fever (RMSF) does not cause visible staining (0/68) or increase the risk of enamel hypoplasia (2/68) (Lochary et al., 1998; Todd et al., 2015). The Centers for Disease Control and Prevention (CDC) strongly recommends the use of doxycycline as the first-line treatment for Rocky Mountain spotted fever (RMSF) in children of all ages, including those under 8 years. The recommended dose for children weighing less than 45 kg is 2.2 mg/kg twice daily, not exceeding 100 mg per dose (CDC, 2024). Both the CDC and the American Academy of Paediatrics (AAP) (Red Book: AAP, 2021), endorse doxycycline for RMSF in young children, prioritizing its life-saving benefits over outdated concerns about dental effects. Despite these strong recommendations, pediatric mortality from RMSF remains higher than that of adults, and some healthcare providers in the United States are still reluctant to prescribe doxycycline to children with suspected RMSF (Dahlgren et al., 2012; Zientek et al., 2014). This review aims to improve healthcare providers’ confidence in their treatment choices and reduce paediatric mortality due to RMSF by providing evidence that the risk of dental staining due to doxycycline is minimal (0/68).

According to paediatric community-acquired pneumonia (CAP) guidelines, doxycycline is recommended for 10 days for infections caused by atypical organisms such as *Mycoplasma* pneumoniae, *Chlamydia trachomatis*, and *Chlamydia* pneumoniae (Bradley et al., 2011). In this review, one study reported no cases of tooth discoloration (0/31) following doxycycline use for respiratory tract infections, including pneumonia, with a treatment duration of 10 days (Volovitz et al., 2007). These findings add to the growing body of evidence that short-term doxycycline use can be safely prescribed to children without concern for tooth discoloration. Nevertheless, high-quality studies are needed to strengthen this evidence base.

In regions like Gorakhpur district in Uttar Pradesh state, India where there is a notable prevalence of scrub typhus encephalitis in children (Mittal et al., 2017), the administration of azithromycin to all fever cases in children has proven effective in reducing scrub typhus encephalitis-related mortality (Thangaraj et al., 2020). Doxycycline could serve as a viable alternative to azithromycin for specific high-risk scenarios where therapeutic alternatives are limited. The apprehension surrounding adverse events associated with doxycycline use in children lacks substantial basis, given the minimal risk compared to the potential benefits and the positive impact it could have on public health.

This review found that tooth discoloration was negligible (1/162), with a median doxycycline administration duration of 8.5 days (IQR: 6–12.5 days). Our findings are consistent with previous reviews, which concluded that doxycycline treatment durations of up to 10 days have negligible effects on tooth staining (Boast et al., 2016; Cross et al., 2016; Stultz and Eiland, 2019; Nieves S et al., 2019).

In addition, this review reports a pooled proportion of adverse events of 0.21 (95% CI: 0.13–0.28). This low proportion does not mean that adverse events do not occur; they are influenced by the dose and duration of doxycycline. The review is limited by the small number of included studies (n = 5), the majority of which are retrospective and lack systematic adverse event reporting, raising concerns about the quality and consistency of the data. These factors restrict the generalizability and applicability of the pooled estimates and underscore the need for well-designed prospective research. Despite the limited number of studies, this review suggests that tooth-related adverse effects from short course of doxycycline are minimal, and concerns may be partially mitigated, though large-scale studies are necessary to thoroughly evaluate the safety.

## Conclusion

This review suggests that the risk of dental-related adverse events associated with short-term doxycycline use in children appears minimal, with a very low proportion of such incidents reported. However, these findings should be interpreted with caution due to several limitations. The small sample size and limited number of eligible studies constrain the robustness and generalizability of the findings, while unclear methodology in one article further limits comparability.

Given these limitations, the current findings should be viewed as preliminary, providing a baseline rather than definitive evidence of doxycycline’s safety in pediatric populations. Nevertheless, they offer important insights into the potential safety of doxycycline use in young children. Many physicians remain hesitant to prescribe doxycycline in this age group due to concerns about dental side effects—even in life-threatening conditions such as Rocky Mountain Spotted Fever, where doxycycline is the only effective treatment. This review contributes to the growing body of evidence suggesting that short courses of doxycycline may be safe, and highlights the need to reconsider current prescribing practices in critical care settings.

The scarcity of large-scale, high-quality studies highlights the need for further research to better understand the long-term impact of doxycycline on dental health and overall safety in children. Future research should focus on large-scale, prospective cohort studies with diverse populations and long-term follow-up to better evaluate the safety profile of doxycycline in pediatric populations. Randomized controlled trials comparing doxycycline with alternative antibiotics are needed to assess relative risks. Special attention should be given to children under the age of 8, with follow-up periods extending to at least 6–12 months post-treatment to adequately monitor for potential adverse effects. Findings from such studies will be critical in guiding evidence-based clinical practice and informing policy decisions regarding the safe use of doxycycline in pediatric populations.

## Data Availability

The data used in this review was derived from publicly available research articles. The dataset used and/or analysed during the current review are available from the corresponding author on reasonable request.
