# Supplementary material for: Dental safety of short-term doxycycline use in children under 8 years: a systematic review and meta-analysis
Source: Front Pharmacol. 2025 Sep 24;16:1646638. doi: 10.3389/fphar.2025.1646638 (PMC12504218; doi:10.3389/fphar.2025.1646638)
Supplement: Supplementary file 1 [file Supplementaryfile1.docx]

**Supplementary files**

**Table of content**

| **Sl.NO** | **Content** | **Page No** |
| --- | --- | --- |
| 1 | **eMethod 1 -** Eligibility criteria in PICOT format for the selection of the studies | 2 |
| 2 | **eMethod 2** - Search strategy used to select the studies | 3 |
| 3 | **eMethod 3 -** Components in data extraction among the included studies for review | 6 |
| 4 | Risk of bias assessment | 8 |
| 5 | List of studies excluded at full-text screening stage, with brief reasons. | 12 |

**eMethod 1 -** Eligibility criteria in PICOT(S) format for the selection of the studies.

| **P**opulation | Children less than 8 years, either gender, of any ethnicity. |
| --- | --- |
| **I**ntervention | Effect of doxycycline |
| **C**omparison | Other drugs or Placebo |
| **O**utcome | Primary Outcome: Safety of doxycycline in children less than 8 years |
| **T**ime | At least 1 year of follow-up for dental staining studies |
| **S**tudy design and setting | Randomized control trials (RCT), case-control, cohort, clinical trial.  Hospital setting. |
| **O**ther inclusion criteria | **Exposure of interest:** Doxycycline dosage and duration of the drug for children aged less than 8 years  **Geographic location of study:** Global  **Language:** English or studies that were translated in English  **Literature type:** Peer-reviewed  **Type of publication:** Original articles |
| **Exclusion criteria** | Allergic to doxycycline or tetracycline  Comparison of different regimens without a placebo/control  Follow-up of patients less than 1 year will be excluded (For dental staining studies)  Abstracts with no full-text available or could not be retrieved, letter to editors, editorials, guidelines, commentary reports, case reports <5 patients.  Animal or cadaveric studies  Other languages |

**eMethod 2 -** Search strategy used to select the studies

**PubMed search**

| **Search number** | **Query** | **Filters** | **Search Details** | **Results** |
| --- | --- | --- | --- | --- |
|  | **((((("doxycycline"[All Fields]) OR ("doxycycline"[MeSH Terms])) OR ("doxycycline"[Title])) OR ("tetracyclines"[Title])) OR ("tetracyclines"[MeSH Terms])) OR ("tetracyclines"[All Fields])** | None | "doxycycline"[All Fields] OR "doxycycline"[MeSH Terms] OR "doxycycline"[Title] OR "tetracyclines"[Title] OR "tetracyclines"[MeSH Terms] OR "tetracyclines"[All Fields] | 65,368 |
|  | **(((((((((((("children"[All Fields]) OR ("child"[MeSH Terms])) OR ("children"[Title])) OR ("pediatric"[Title])) OR ("pediatric"[All Fields])) OR ("pediatric age"[All Fields])) OR ("pediatric age"[Title])) OR ("under five"[Title])) OR ("under five age"[Title])) OR ("under five age children"[Title])) OR ("under five age group"[Title])) OR ("young children"[Title])) OR ("young children"[All Fields])** | None | "children"[All Fields] OR "child"[MeSH Terms] OR "children"[Title] OR "pediatric"[Title] OR "pediatric"[All Fields] OR "pediatric age"[All Fields] OR "pediatric age"[Title] OR "under five"[Title] OR "under five age"[Title] OR "under five age children"[Title] OR "under five age group"[Title] OR "young children"[Title] OR "young children"[All Fields] | 3,06,732 |
|  | **(((((("safety"[All Fields]) OR ("safety"[MeSH Terms])) OR ("safety"[Title])) OR ("efficacy"[Title])) OR ("efficacy"[All Fields])) OR ("effectiveness"[All Fields])) OR ("effectiveness"[Title])** | None | "safety"[All Fields] OR "safety"[MeSH Terms] OR "safety"[Title] OR "efficacy"[Title] OR "efficacy"[All Fields] OR "effectiveness"[All Fields] OR "effectiveness"[Title] | 2,225,471 |
|  | **((((("adverse effects"[All Fields]) OR ("adverse effects"[Title])) OR ("side effects"[Title])) OR ("side effects"[All Fields])) OR ("adverse events"[All Fields])) OR ("adverse events"[Title])** | None | "adverse effects"[All Fields] OR "adverse effects"[Title] OR "side effects"[Title] OR "side effects"[All Fields] OR "adverse events"[All Fields] OR "adverse events"[Title] | 2,462,031 |
|  | **#1 AND #2** | None | ("doxycycline"[All Fields] OR "doxycycline"[MeSH Terms] OR "doxycycline"[Title] OR "tetracyclines"[Title] OR "tetracyclines"[MeSH Terms] OR "tetracyclines"[All Fields]) AND ("children"[All Fields] OR "child"[MeSH Terms] OR "children"[Title] OR "pediatric"[Title] OR "pediatric"[All Fields] OR "pediatric age"[All Fields] OR "pediatric age"[Title] OR "under five"[Title] OR "under five age"[Title] OR "under five age children"[Title] OR "under five age group"[Title] OR "young children"[Title] OR "young children"[All Fields]) | 4,665 |
|  | **#1 AND #3 AND #4** | None | ("doxycycline"[All Fields] OR "doxycycline"[MeSH Terms] OR "doxycycline"[Title] OR "tetracyclines"[Title] OR "tetracyclines"[MeSH Terms] OR "tetracyclines"[All Fields]) AND ("safety"[All Fields] OR "safety"[MeSH Terms] OR "safety"[Title] OR "efficacy"[Title] OR "efficacy"[All Fields] OR "effectiveness"[All Fields] OR "effectiveness"[Title]) AND ("adverse effects"[All Fields] OR "adverse effects"[Title] OR "side effects"[Title] OR "side effects"[All Fields] OR "adverse events"[All Fields] OR "adverse events"[Title]) | 1,571 |
|  | **#5 AND #6** | None | ("doxycycline"[All Fields] OR "doxycycline"[MeSH Terms] OR "doxycycline"[Title] OR "tetracyclines"[Title] OR "tetracyclines"[MeSH Terms] OR "tetracyclines"[All Fields]) AND ("children"[All Fields] OR "child"[MeSH Terms] OR "children"[Title] OR "pediatric"[Title] OR "pediatric"[All Fields] OR "pediatric age"[All Fields] OR "pediatric age"[Title] OR "under five"[Title] OR "under five age"[Title] OR "under five age children"[Title] OR "under five age group"[Title] OR "young children"[Title] OR "young children"[All Fields]) AND (("doxycycline"[All Fields] OR "doxycycline"[MeSH Terms] OR "doxycycline"[Title] OR "tetracyclines"[Title] OR "tetracyclines"[MeSH Terms] OR "tetracyclines"[All Fields]) AND ("safety"[All Fields] OR "safety"[MeSH Terms] OR "safety"[Title] OR "efficacy"[Title] OR "efficacy"[All Fields] OR "effectiveness"[All Fields] OR "effectiveness"[Title]) AND ("adverse effects"[All Fields] OR "adverse effects"[Title] OR "side effects"[Title] OR "side effects"[All Fields] OR "adverse events"[All Fields] OR "adverse events"[Title])) | 181 |

**Cochrane search**

**#1** MeSH descriptor: [Doxycycline] explode all trees **1233**

**#2** MeSH descriptor: [Pediatrics] explode all trees **1179**

**#3** MeSH descriptor: [Safety] explode all trees **18473**

**#4** MeSH descriptor: [Treatment Outcome] explode all trees **182794**

**#5** (doxycycline OR tetracycline):ti,ab,kw (Word variations have been searched**) 4646**

**#6** (doxycycline OR tetracycline):ti,ab,kw AND ("pediatric" OR children OR "Under five"):ti,ab,kw (Word variations have been searched) **357**

**Other searches:** Google scholar

**eMethod 3 -** Components in data extraction among the included studies for review

The data extraction form includes,

1. First author name
2. Publication year
3. Journal name
4. Objective of the study
5. Study design
6. Randomization technique, if RCT
7. Allocation concealment, if RCT
8. Study setting (hospital or community)
9. Study period (in weeks)
10. Blinding status (Single or double or triple blinding)
11. Study country
12. Sample size
13. Study population (inclusion criteria)
14. Age (mean ± SD or median (IQR)) (in years)
15. Gender
16. Details of the antibiotic Doxycycline – dose (mg), route, frequency (OD or BD), and duration of administration, and any other specific data.
17. Disease under study
18. Type of analysis (PP/ITT)
19. Placebo or control drug details (drug name, dose (mg), route, frequency (OD or BD), and duration of administration)
20. Any comorbidities
21. Presence or absence of any background therapy
22. Method of assessment
23. Adverse events
24. Overall results
25. Limitations

**Risk of bias assessment**


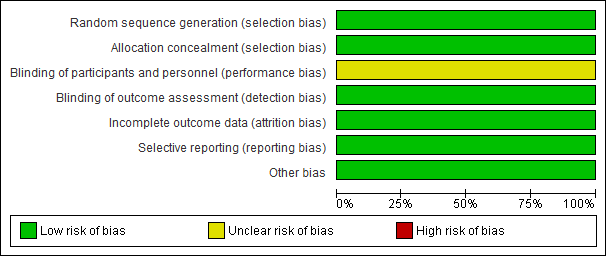


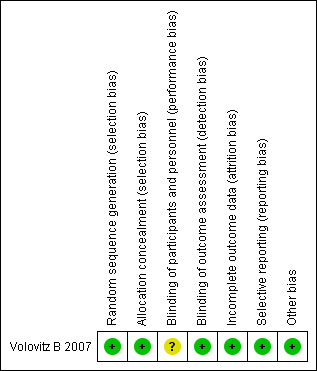


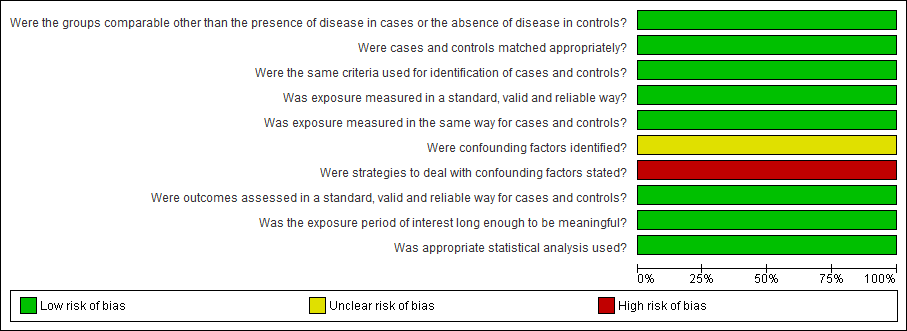


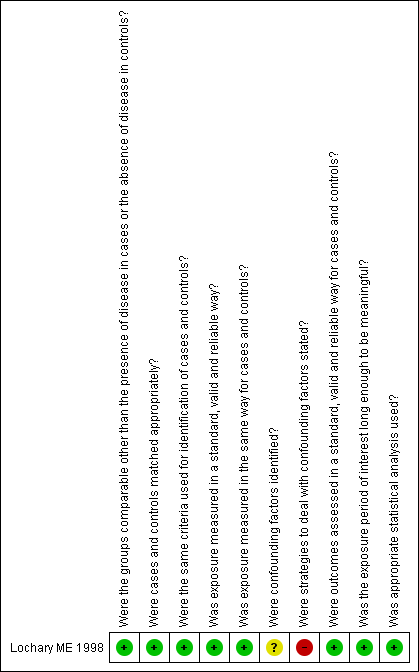


**
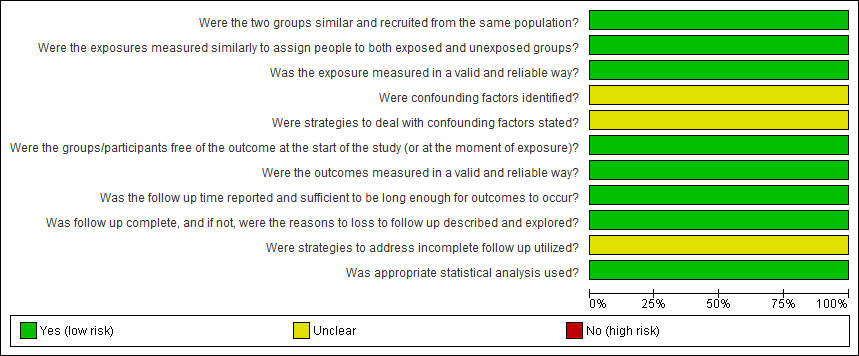
**


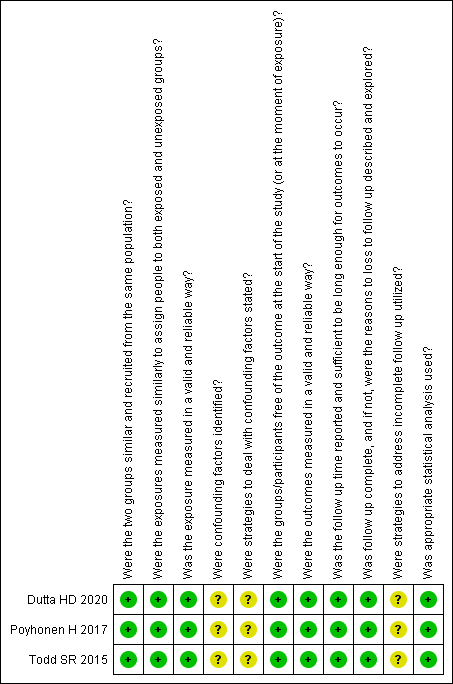

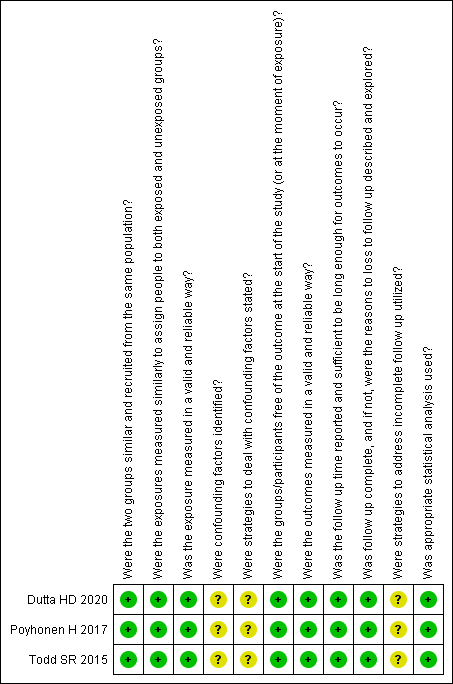


**List of studies excluded at full-text screening stage**

| **Reason** | **Number of studies excluded** |
| --- | --- |
| Total Studies excluded | 105 |
| Language | 2 |
| Patient population | 45 |
| Full text not available | 5 |
| Study design | 39 |
| Wrong Outcomes | 11 |
| Wrong Intervention | 3 |
